# Radiomics analysis of pericoronary adipose tissue for detecting ischaemia with non-obstructive coronary arteries in NAFLD patients

**DOI:** 10.1186/s12872-025-05292-5

**Published:** 2025-11-12

**Authors:** Lingli Wang, Hongming Luo, Yuanbo Xiong, Kaixiang Su, Siyu Jiang, Guangju Zhou, Rui Li

**Affiliations:** 1 Department of Radiology, Affiliated Hospital of North Sichuan Medical College and Sichuan Key Laboratory of Medical Imaging, Nanchong, China; 2 Department of Radiology, Chengdu Fifth People’s Hospital, Chengdu, China; 3 Department of Radiology, Ziyang Central Hospital, Ziyang, China; 4 School of Medicine, Jiangsu University, Zhenjiang, China; 5 Department of Endocrinology, Affiliated Hospital of North Sichuan Medical College, Nanchong, China

**Keywords:** Nonalcoholic fatty liver disease, Ischaemia with non-obstructive coronary arteries, Pericoronary adipose tissue, Radiomics feature, Vascular inflammation

## Abstract

**Background:**

Chronic low-grade inflammation in nonalcoholic fatty liver disease (NAFLD) plays a critical role in the development of cardiovascular complications, particularly ischaemia with non-obstructive coronary arteries (INOCA). This study aimed to develop and evaluate models combining pericoronary adipose tissue (PCAT) radiomics, PCAT attenuation (PCATa), CCTA plaque parameters, and clinical risk factors to identify INOCA in NAFLD patients.

**Methods:**

This retrospective study included 159 patients with NAFLD who underwent CCTA. The patients were randomly divided into the training (70%) and validation (30%) cohorts. Clinical features, CCTA imaging indicators, and right coronary artery PCAT radiomic features were analyzed. Five models were constructed using logistic regression: Model 1 (PCATa model), Model 2 (radiomics model), Model 3 (clinical factors model), Model 4 (combined imaging model), and Model 5 (combined imaging-clinical model). The models’ diagnostic performance was assessed using the area under the curve, reclassification metrics, and decision curve analysis (DCA).

**Results:**

The PCAT radiomics model exhibited higher diagnostic efficacy than the PCATa model in identifying INOCA (training cohort: AUC 0.734 vs. 0.674; validation cohort: AUC 0.706 vs. 0.637). The combined imaging model showed improved performance over the clinical factors model (training AUC 0.830, validation AUC 0.813). The model integrating imaging and clinical factors achieved the highest diagnostic accuracy (AUCs of 0.873 and 0.824 in the training and validation cohorts, respectively), demonstrating incremental value based on improved NRI, IDI, and DCA metrics. Calibration analysis indicated good agreement between predicted and observed outcomes.

**Conclusions:**

The radiomics model provided better discrimination than the PCATa model for identifying INOCA among patients with NAFLD. Models incorporating radiomics and CCTA imaging parameters outperformed those based solely on clinical factors. The comprehensive imaging-clinical model achieved the best overall performance and may serve as a promising non-invasive approach for INOCA risk stratification in NAFLD, although external validation is still required.

**Supplementary Information:**

The online version contains supplementary material available at 10.1186/s12872-025-05292-5.

## Background

 Nonalcoholic fatty liver disease (NAFLD), the most prevalent chronic liver disease worldwide, has been reported in a recent systematic review to have an Asian prevalence of approximately 29.62%, with an annual increasing trend over time [1]. In addition to hepatic manifestations, NAFLD is closely associated with various extrahepatic complications, particularly cardiovascular diseases. Studies have shown that cardiovascular diseases have become the leading cause of mortality in patients with NAFLD [2, 3]. Notably, even with effective control of traditional cardiovascular risk factors, the incidence of coronary artery disease (CAD) remains significantly elevated, particularly with non-obstructive CAD being more prominent in this population [4]. Non-obstructive CAD, typically defined as coronary artery stenosis of less than 50%, has complex pathophysiological mechanisms involving inflammation, plaque vulnerability, and microvascular dysfunction [5]. Despite the absence of significant coronary stenosis on coronary computed tomography angiography (CCTA), some patients exhibit symptoms of myocardial ischemia, a condition referred to as ischemia with non-obstructive coronary arteries (INOCA) [6]. This condition has gained increasing recognition in recent years as a substantial contributor to adverse cardiovascular outcomes and mortality [7]. Therefore, developing novel diagnostic models to improve the early detection of INOCA in NAFLD patients is crucial for the proactive prevention and management of CAD progression.

Recent studies have revealed that in NAFLD, because of prolonged exposure to chronic low-grade inflammation [8], inflammatory mediators that can induce coronary artery inflammatory response are released. This inflammation may be closely associated with the onset and progression of adverse cardiovascular outcomes [9]. When coronary arteries become inflamed, inflammatory factors diffuse outward from the intima, altering the adjacent pericoronary adipose tissue (PCAT) [10, 11], leading to changes in PCAT attenuation (PCATa) and volume, reported by several studies that patients with NAFLD usually exhibit higher PCATa [12]. CCTA effectively evaluates the degree of coronary artery stenosis and plaque parameters [13], offering quantitative insights into PCAT changes and aiding in the identification of underlying vascular inflammation and disease activity. However, vascular inflammation can further induce irreversible changes in adipose tissue structure, such as microvascular remodeling and fibrosis [14–16], which cannot be captured by measuring PCAT alone. In contrast, PCAT radiomics analysis based on CCTA can identify microstructural features and persistent changes in the perivascular space caused by inflammation, offering valuable insights beyond PCATa [17]. Therefore, integrating CCTA with radiomics to analyze PCAT characteristics in patients with NAFLD may help identify high-risk individuals for non-obstructive CAD and provide a basis for early intervention.

Although previous studies have established a strong association between PCAT inflammation and the development of CAD, research on the radiomic characteristics of PCAT in patients with NAFLD remains limited. In particular, the use of radiomics to extract quantitative PCAT features from CCTA images and integrate them into the diagnosis of INOCA has not been extensively explored. Therefore, this study developed a comprehensive model integrating clinical data, CCTA imaging parameters, and PCAT radiomic features to identify risk factors associated with INOCA in NAFLD patients. By focusing on coronary inflammation and plaque characteristics, this approach aims to improve the early diagnosis of INOCA in patients with NAFLD and to provide an imaging-based reference for identifying high-risk patients with non-obstructive CAD, thereby enabling timely clinical intervention and management.

## Method

### Study population

**Fig. 1:**
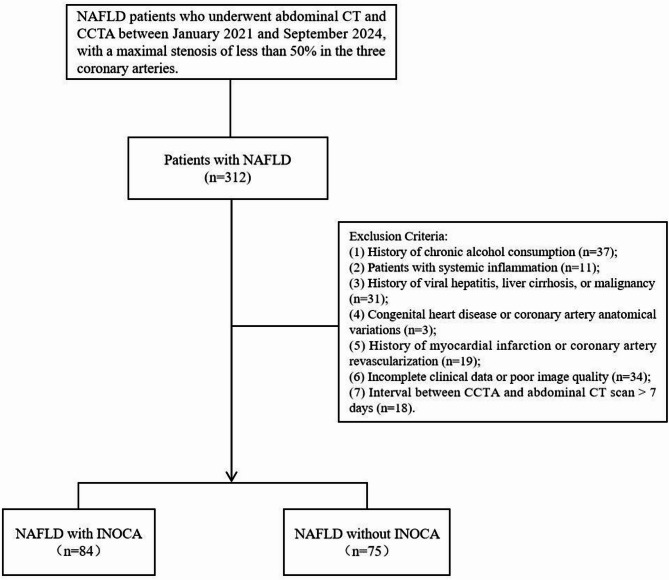
Flow chart showing the selection process for the study population. CCTA, coronary computed tomography angiography; INOCA, ischaemia with non-obstructive coronary arteries; NAFLD, nonalcoholic fatty liver disease

This study was conducted in accordance with the Declaration of Helsinki and was approved by the Ethics Committee of the Affiliated Hospital of North Sichuan Medical College (No. 2024ER39-1). The requirement for informed consent was waived due to the retrospective nature of the study. This retrospective study examined NAFLD patients who underwent CCTA at our institution between January 2021 and September 2024. Inclusion criteria: (1) Patients who underwent CCTA, with maximal stenosis of less than 50% in the three major coronary arteries. (2) Patients with a prior diagnosis of NAFLD and those newly diagnosed during their visit, defined as individuals who underwent non-contrast-enhanced abdominal CT within 3 days before or after CCTA and had a clinically confirmed diagnosis of NAFLD [18]. Exclusion criteria: (1) history of moderate to excessive alcohol consumption; (2) systemic inflammatory diseases; (3) history of viral hepatitis, liver cirrhosis, or malignancies; (4) congenital heart disease or coronary artery anatomical variations; (5) history of myocardial infarction or coronary revascularization; (6) incomplete clinical data or poor image quality; (7) interval between coronary CTA and abdominal CT scan >7 days. Ultimately, 159 patients with NAFLD were recruited in the study, of whom 84 were clinically diagnosed with INOCA, whereas the remaining 75 did not have CAD. A separate group of 166 patients without NAFLD was selected as the control group to ensure comparability with the NAFLD group in terms of age, sex, cardiovascular risk factors, and CCTA acquisition parameters. Figure 1 presents the study design workflow.

### Identification of INOCA in patients

Accurate diagnosis of INOCA has traditionally required both structural coronary angiography to exclude obstructive coronary artery disease and functional coronary angiography to evaluate underlying pathophysiological mechanisms [19, 20]. However, despite the ability of functional coronary angiography to identify the key physiological mechanisms responsible for ischemic chest pain in INOCA patients, only a minority of patients with chest pain and non-obstructive CAD currently undergo such evaluation. Therefore, following previously described methodologies and a review published in the BMJ, it is recommended that once non-cardiac and non-ischemic causes of chest pain have been excluded, the diagnosis of INOCA should be considered in patients with evidence of myocardial ischemia—even if the underlying mechanism is not definitively established [20, 21]. The diagnostic criteria primarily include the following: typical angina symptoms (which may occur during exertion, stress, or at rest); ischemic changes on electrocardiography; stress-induced myocardial perfusion defects or regional wall motion abnormalities; and impaired coronary flow reserve. If any of these indicators of myocardial ischemia are present and the degree of coronary artery stenosis is less than 50%, a diagnosis of INOCA can be considered.

### CT assessment of NAFLD

**Fig. 2:**
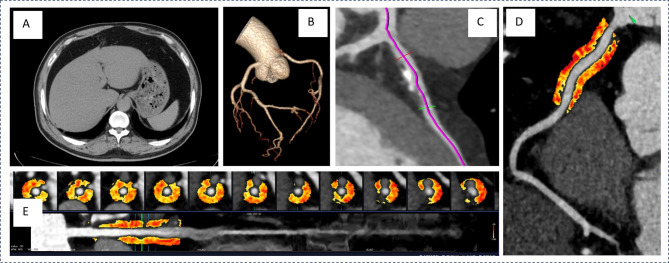
Illustration of Abdominal CT and CCTA Measurement Parameters in a Typical Patient. **A** Abdominal CT scan confirming NAFLD diagnosis through liver and spleen measurements. **B** Reconstructed coronary artery bundle from CCTA. **C** Coronary Artery Plaque Measurement Diagram. **D** Diagram illustrating PCAT parameter measurements for the proximal 40-mm segment of coronary arteries. **E** Regions of interest for radiomic analysis and the proximal slice of PCAT under direct visualization. CCTA, coronary computed tomography angiography; PCAT, pericoronary adipose tissue; NAFLD, non-alcoholic fatty liver disease

The evaluation of NAFLD was performed by measuring the CT attenuation values of the liver and spleen on non-contrast CT imaging. Circular regions of interest, each covering at least 2 × 2 cm, were selected on three cross-sectional slices of the liver and spleen [22]. The average Hounsfield unit (HU) values were recorded, and the liver-to-spleen attenuation ratio was calculated, with a ratio of < 1.0 serving as the diagnostic threshold for fatty liver [23] **(**Fig. 2A**)**. The cases were identified as NAFLD after excluding other potential causes of hepatic steatosis. Two radiologists independently performed the measurements, and in instances of significant discrepancies, a third experienced radiologist reviewed the images to provide the final determination.

### CCTA acquisition

All CCTA scans were performed using a third-generation dual-source CT scanner (Somatom Force; Siemens Healthineers, Germany). The patients underwent pre-scan preparation, including heart rate control and breathing exercises. For those with a heart rate exceeding 80 bpm, metoprolol (25–100 mg) was administered to reduce the heart rate below 80 bpm. After achieving the target heart rate and confirming that there were no contraindications for CCTA, the patients were positioned supine, and electrodes were placed on the left anterior chest for ECG gating. The ECG signal was calibrated to ensure clear R-wave visibility for accurate scan synchronization. The patients were positioned in a supine head-first orientation, and scanning was performed using prospective ECG-triggered acquisition during the end-expiratory breath-hold. The scan parameters were as follows: rotation time = 280 ms, trigger window covering 35%–75% of the R-R interval, collimation = 256 × 0.625 mm, reconstructed slice thickness = 0.625 mm, and reconstruction interval = 0.5 mm. The tube potential (70–120 kV) was adjusted based on the patient’s body size and clinical needs, with automatic exposure control, thereby optimizing the balance between image quality and radiation dose. Images were reconstructed using smooth kernel (STANDARD) and iterative reconstruction techniques (50% strength, ASiR-V, GE Healthcare). Diastolic-phase images were used for CCTA analysis to ensure precision and clarity.

### CCTA imaging parameters

The CCTA plasmon parameters were transferred to a Shukun workstation (Shukun Technology Co., Ltd, Beijing, China) for evaluation. The CCTA data were consistently analyzed by two experienced radiologists using the CAD Reporting and Data System. Each coronary segment was assessed, and segments with a diameter < 2 mm, severe motion artifacts, or low contrast resolution were excluded from further analysis. The software automatically generated scan-specific threshold values for plaque composition. An adaptive algorithm was used to quantify plaque components within manually designated regions. The location, volume, total plaque volume(TPV), and diameter stenosis(DS) severity of the three major coronary arteries were recorded. In this study, the most severe diameter stenosis (DS_max_) among the three coronary arteries was selected for analysis. Each plaque component was automatically identified and quantified by the software, with manual adjustments made as necessary (Fig. 2 B and C).

PCAT measurements were performed for all participants using CoronaryDoc (version 6.21; Shukun Technology Co., Ltd, Beijing, China). PCATa was defined as the mean CT attenuation of adipose tissue within a radial distance equivalent to the diameter of the target vessel, measured from the outer vessel wall. The software automatically tracked the proximal 40-mm segments of the three coronary arteries, including the right coronary artery (RCA), left anterior descending artery (LAD), and left circumflex artery (LCX), during the diastolic phase. Only voxels with attenuation values between − 190 and − 30 HU were analyzed [24, 25] (Fig. 2 D). The PCAT volumes of the proximal 40-mm segments of all three coronary arteries were recorded. To account for the effects of tube voltage on PCATa, the HU values were corrected for each tube voltage setting (70 kV, 80 kV, 90 kV, 100 kV, and 110 kV) by dividing them by the corresponding coefficients, as previously reported [26]. The corrected PCATa values were then used in subsequent analyses.

Furthermore, radiomic features were automatically extracted from the PCAT using the CoronaryDoc (version 6.21; Shukun Technology Co., Ltd, Beijing, China), as previously described (Fig. 2 E). The designated cross-sectional area for analysis adhered to the previously established standards for measuring PCATa [24]. All preprocessing procedures, including voxel resampling, intensity normalization, and gray-level discretization, were performed according to the software’s standardized radiomics pipeline. In total, ninety-five radiomic features were subsequently extracted from each PCAT using CoronaryDoc, including morphological, first-order histogram, and higher-order texture features, thereby providing a comprehensive characterization of the PCAT for analysis.

### Feature selection and prediction model development

**Fig. 3:**
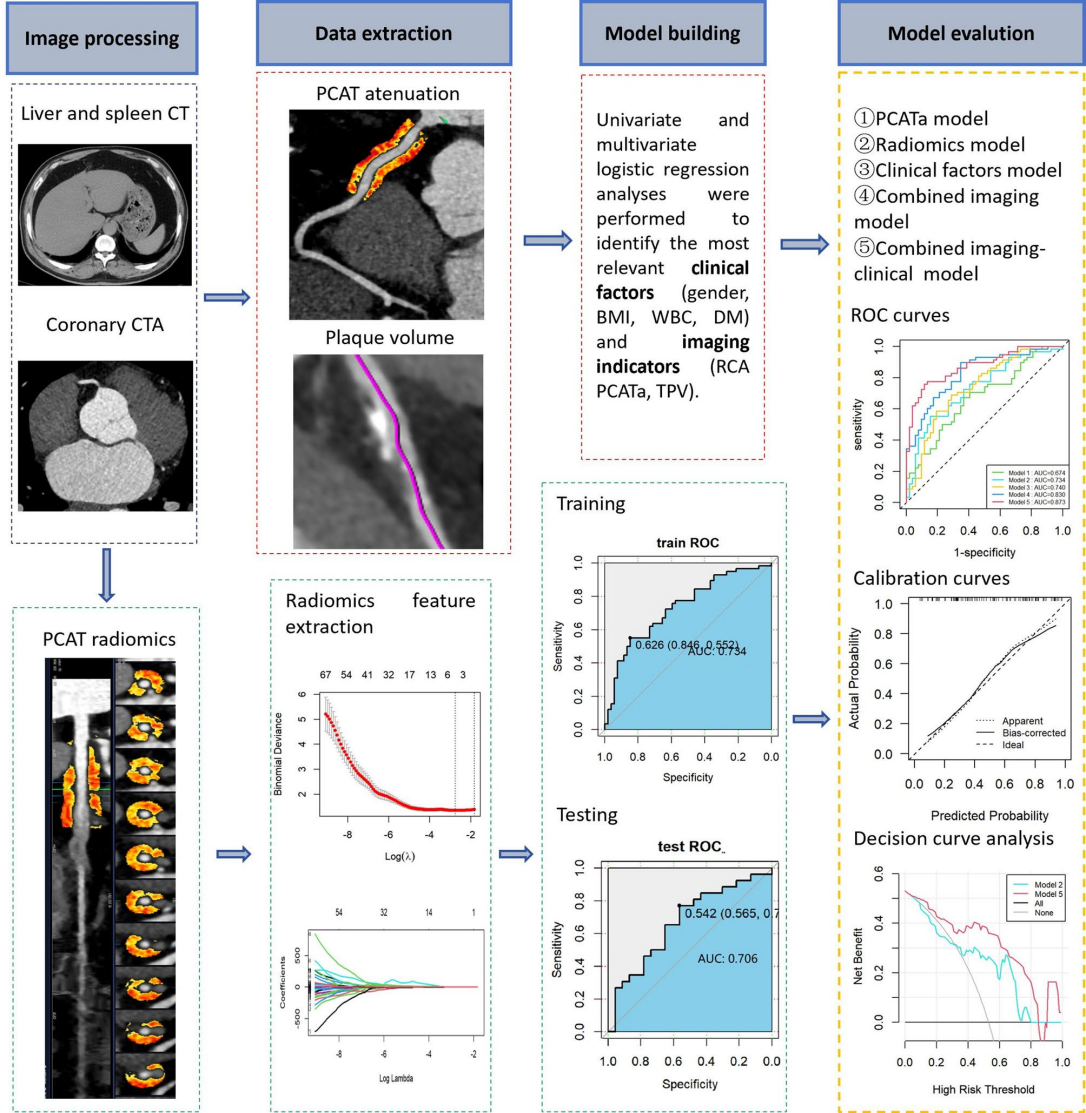
Flowchart illustrating the development of the integrated radiomics-based model. CT, computed tomography; CTA, computed tomography angiography; PCATa, pericardial adipose tissue attenuation; RCA, right coronary artery; AUC, area under curve; BMI, body mass index; WBC, white blood cell count; DM, diabetes mellitus

The 95 radiomic features were first standardized using z-score normalization. The dataset was randomly divided into training and validation cohorts at a ratio of 7:3. Feature selection was performed in a stepwise manner: features with significant differences between groups were first identified using univariate testing (t-test or Mann–Whitney U, *p* < 0.05), and highly correlated features (Spearman |ρ| >0.90) were removed to reduce redundancy. The least absolute shrinkage and selection operator (LASSO) logistic regression with tenfold cross-validation was then applied to the remaining features to determine the optimal λ value and avoid overfitting. This process yielded two radiomic features with nonzero coefficients (Cluster Prominence and Low Gray Level Run Emphasis), which were used for model fitting and Rad-score calculation (Supplementary Table S3). The radiomic features were further incorporated into other models to construct a combined predictive model. In parallel, univariate logistic regression identified four clinical factors and two imaging indices, which were integrated with the radiomics signature to develop the final combined model. Five generalized logistic regression models were developed, and their performance in differentiating patients with NAFLD and INOCA from those without CAD was evaluated: Model 1: PCATa; Model 2: radiomics; Model 3: clinical factors; Model 4: combined imaging indicators; and Model 5: combined imaging-clinical model. Figure 3 presents the study workflow.

### Statistical analysis

All statistical analyses were performed using Statistical Package for the Social Sciences (version 26.0; Armonk, NY, USA) and R Studio (version 4.3.3). Continuous variables are expressed as means ± standard deviations or medians (interquartile ranges [IQRs], Q25–75), whereas categorical variables are presented as percentages. Continuous variables were compared using independent Student’s *t*-test or the Mann–Whitney U test. The chi-square test was used to compare categorical variables. *P*-values < 0.05 were used to denote statistical significance. Univariate and multivariate logistic regression analyses were performed to identify the independent factors influencing CAD in patients with NAFLD. The performance of the five predictive models was assessed for discrimination, calibration, and clinical utility. Receiver operating characteristic (ROC) curve analysis was performed to evaluate the diagnostic efficacy of the models, and area under the ROC curve (AUC) values were compared using the DeLong test. The reclassification performance was analyzed using the integrated discrimination improvement (IDI) and net reclassification improvement (NRI) indices. Calibration curves were used to compare the predicted probabilities with the observed outcomes for the combined clinical-imaging model in the training and validation cohorts. Decision curve analysis (DCA) was performed to quantify the clinical utility of the models by measuring the net benefit across different threshold probabilities.

## Result

### Baseline characteristics

This study included 159 patients with NAFLD and 166 patients without NAFLD. Table 1 presents the baseline characteristics of the patients. The NAFLD group had a mean age of 63.04 ± 11.44 years, with 66.67% of the patients being male. The NAFLD group exhibited significantly higher TC, TG, LDL, ALT, BMI, and WBC levels and lower HDL levels than the non-NAFLD group (all *p* < 0.05). The NAFLD group was further divided into two subgroups according to the presence of INOCA. The INOCA group was more likely to be male (*p* < 0.001), had a higher prevalence of diabetes mellitus (*p* = 0.007), exhibited higher BMI (*p* = 0.011) and ALT levels (*p* < 0.001), and had higher levels of inflammatory markers, such as WBC, NLR, and PLR than the non-INOCA group (all *p* < 0.05). Interestingly, TC levels were lower in the INOCA group than in the non-INOCA group (*p* = 0.002).

**Table 1:** Comparison of baseline characteristics among groups

	Non-NAFLD *N* = 166	NAFLD *N* = 159	*P* value	NAFLD without INOCA *N* = 75	NAFLD with INOCA *N* = 84	*P* value
Clinical characteristics						
Age(y)	63.80 ± 6.92	63.04 ± 11.44	0.470	61.89 ± 11.24	64.07 ± 11.59	0.232
Gender (male), n(%)	82 (49.40)	86 (54.09)	0.398	30 (40.00)	56 (66.67)	< 0.001
BMI, kg/m^2^	23.05 (21.51, 24.42)	26.67 (24.40, 29.11)	< 0.001	25.88 (23.38, 27.70)	26.90 (25.19, 30.10)	0.011
Hypertension, n (%)	84 (50.60)	92 (57.86)	0.189	38 (50.67)	54 (64.29)	0.083
Diabetes, n (%)	53 (31.93)	58 (36.48)	0.387	19 (25.33)	39 (46.43)	0.006
History of smoking, n (%)	46 (27.71)	54 (33.96)	0.222	21 (28.00)	33 (39.29)	0.134
Lipids						
AST, U/L	22.00 (19.00, 26.75)	23.00 (19.00, 33.00)	0.118	22.00 (18.00, 30.00)	23.00 (19.00, 34.25)	0.284
ALT, U/L	20.50 (15.00, 32.75)	24.00 (16.50, 37.00)	0.020	19.00 (14.00, 30.00)	28.50 (19.75, 45.25)	< 0.001
AST/ALT	1.11 (0.79, 1.44)	1.05 (0.77, 1.31)	0.114	1.05 (0.83, 1.32)	1.03 (0.74, 1.28)	0.338
TC, mmol/L	4.27 (3.88, 4.83)	4.81 (4.09, 5.38)	< 0.001	5.04 (4.48, 5.56)	4.57 (3.73, 5.37)	0.002
TG, mmol/L	1.11 (1.00, 1.56)	2.05 (1.42, 3.06)	< 0.001	2.03 (1.52, 2.82)	2.06 (1.41, 3.10)	0.786
LDL, mmol/L	2.37 (1.85, 2.77)	2.70 (2.11, 3.31)	0.002	2.77 (2.27, 3.41)	2.66 (1.87, 3.18)	0.129
HDL, mmol/L	1.15 (1.01, 1.41)	1.04 (0.89, 1.21)	< 0.001	1.04 (0.92, 1.27)	1.02 (0.88, 1.16)	0.059
Inflammatory markers						
White Blood Cell Count, *10^9^/L	5.53 (4.82, 6.80)	6.63 (5.68, 8.25)	< 0.001	6.16 (5.19, 7.38)	7.06 (6.19, 8.92)	0.006
C-reactive protein, mg/L	1.16 (0.76, 3.23)	3.67 (1.27, 7.44)	< 0.001	3.67 (1.12, 6.57)	3.67 (1.38, 7.85)	0.296
NLR	2.22 (1.61, 3.11)	2.47 (1.78, 3.37)	0.124	2.01 (1.56, 2.94)	2.70 (2.14, 3.71)	< 0.001
PLR	114.68 (85.29, 147.71)	111.63 (87.82, 144.99)	0.880	102.00 (80.03, 124.65)	128.80 (98.14, 154.96)	0.001
Medication situation						
ACEI/ARB	14 (8.43)	18 (11.32)	0.383	9 (12.00)	9 (10.71)	0.798
β-blocker	8 (4.82)	11 (6.92)	0.420	3 (4.00)	8 (9.52)	0.171
CCBs	20 (12.05)	28 (17.61)	0.158	9 (12.00)	19 (22.62)	0.079
Statins	31 (18.67)	45 (28.30)	0.040	19 (25.33)	26 (30.95)	0.432
PCAT attenuation, HU						
LAD	−82.27 ± 8.50	−79.90 ± 6.65	0.005	−81.05 ± 6.80	−78.88 ± 6.38	0.039
LCX	−75.30 ± 6.71	−73.21 ± 7.09	0.007	−74.36 ± 6.42	−72.18 ± 7.53	0.053
RCA	−82.38 ± 8.40	−78.45 ± 7.58	< 0.001	−80.28 ± 7.40	−76.82 ± 7.41	0.004
PCAT volume, mm^3^						
LAD	1929.48 ± 486.79	1974.26 ± 446.19	0.388	2014.20 ± 491.22	1938.61 ± 401.40	0.293
LCX	1306.96 ± 421.60	1313.83 ± 450.23	0.887	1337.26 ± 475.15	1292.91 ± 428.55	0.537
RCA	2150.94 ± 576.72	2236.83 ± 570.09	0.178	2296.97 ± 598.34	2183.13 ± 541.57	0.210
DS, n (%)						
LAD	-	0.00 (0.00, 28.00)	-	0.00 (0.00, 17.50)	11.00 (0.00, 40.25)	< 0.001
LCX	-	0.00 (0.00, 15.00)	-	0.00 (0.00, 0.00)	0.00 (0.00, 25.00)	< 0.001
RCA	-	16.00 (0.00, 28.00)	-	0.00 (0.00, 19.50)	25.00 (11.75, 34.25)	< 0.001
DS_max_	-	22.00 (4.00, 39.50)	-	11.00 (0.00, 25.50)	33.00 (16.00, 45.00)	< 0.001
TPV, mm^3^	-	55.48 (6.50, 96.23)	-	27.36 (0.00, 59.92)	81.92 (42.62, 125.21)	< 0.001

Notes: *P *values obtained from univariate analysis were used to evaluate the associations among various variables, indicating statistically significant differences

Abbreviations: *INOCA* ischaemia with non-obstructive coronary arteries, *BMI* body mass index, *AST* aspartate aminotransferase, *ALT* alanine aminotransferase, *TC* total cholesterol, *TG* triglycerides, *LDL* low-density lipoprotein, *HDL* high-density lipoprotein, *NLR* neutrophil-to-lymphocyte ratio, *PLR* platelet-to-lymphocyte ratio, *ACEI* angiotensin-converting enzyme inhibitors, *ARB* angiotensin receptor blockers, *β-blocker* beta-blocker, *PCAT* pericoronary adipose tissue, *DS* diameter stenosis, *TPV* total plaque volume, *LAD* left anterior descending artery, *LCX * left circumflex artery, *RCA* right coronary artery

In terms of imaging parameters, the NAFLD group exhibited significantly higher PCATa values in all three major coronary arteries compared to the non-NAFLD group (LAD: −82.27 ± 8.50 HU vs. −79.90 ± 6.65 HU, *p* = 0.005; LCX: −75.30 ± 6.71 HU vs. −73.21 ± 7.09 HU, *p* = 0.007; RCA: −82.38 ± 8.40 HU vs. −78.45 ± 7.58 HU, *p* < 0.001). Among NAFLD patients, those with INOCA exhibited significantly higher LAD and RCA-PCAT attenuation values (LAD: −78.88 ± 6.38 HU vs. −81.05 ± 6.80 HU, *p* = 0.039; RCA: −76.82 ± 7.41 HU vs. −80.28 ± 7.40 HU, *p* = 0.004) and lower TPV and DS_max_ (*p* < 0.001) compared to those without INOCA. However, no statistically significant differences in the proximal PCAT volumes of the three coronary arteries were observed between the groups (*p* > 0.05). Supplemental Table 1 presents the clinical and imaging characteristics of patients in the training and testing cohorts. No significant correlation was observed between variables and INOCA occurrence in either cohort.

### Feature selection and prediction model development

**Fig. 4:**
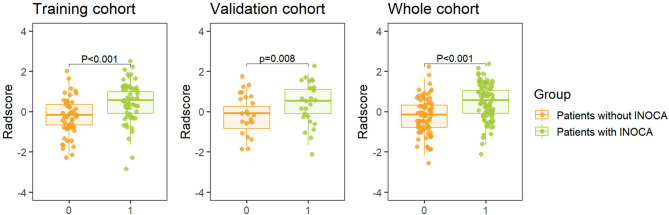
Radscore of NAFLD Patients with INOCA or Without INOCA Across Cohorts. INOCA, ischaemia with non-obstructive coronary arteries; NAFLD, nonalcoholic fatty liver disease

Univariate logistic regression was used to evaluate the clinical and imaging parameters associated with INOCA, with variables showing statistical significance (*p* < 0.05) included in the multivariate logistic regression analysis. Using a forward stepwise approach, male, diabetes mellitus, WBC count, and BMI were independent predictors of INOCA (all *p* < 0.05) **(**Table 2**)**. These four clinical indicators were used to construct a clinical feature model (Model 3) *via* logistic regression. Similarly, univariate and multivariate analyses confirmed that RCA-PCATa and TPV were significantly associated with INOCA. The PCATa model (Model 1) was constructed using the RCA-derived PCATa, as described above. In our analysis, only RCA-PCAT attenuation was significantly associated with INOCA, whereas LAD- and LCX-PCAT attenuation did not reach statistical significance. Considering that the RCA is less frequently affected by calcification and motion artifacts compared with the LAD and LCX, which allows more consistent segmentation and reproducible measurement, the proximal RCA-PCAT region was therefore used as the primary site for feature extraction in the radiomic analysis. The LASSO regression analysis identified two significant radiomic features with nonzero coefficients (Cluster Prominence and Low Gray Level Run Emphasis) as important predictors. These features were used to construct the radiomics model (Model 2), and the Radscore was calculated based on these features. The Radscore differed significantly between INOCA and non-INOCA groups (*p* < 0.05; Fig. 4). Furthermore, a combined imaging model (Model 4) was developed by integrating imaging indicators (PCATa, TPV) with radiomic features. A combined clinical-imaging model (Model 5) was constructed by combining clinical factors, imaging indicators, and radiomic features. These models were subsequently used for the diagnostic assessment of INOCA in patients with NAFLD.

**Table 2:** Univariable and multivariable logistic regression analyses for INOCA

	Univariable Analysis		Multivariable Analysis	
	OR (95% CI)	*P* value	OR (95% CI)	*P* value
Age(y)	1.017 (0.989 ~ 1.045)	0.231		
Gender (male)	3.000 (1.570 ~ 5.732)	< 0.001	2.751 (1.330 ~ 5.693)	0.007
BMI, kg/m^2^	1.116 (1.022 ~ 1.218)	0.015	1.116 (1.008 ~ 1.235)	0.034
Hypertension	1.753 (0.928 ~ 3.310)	0.084		
Diabetes	2.554 (1.302 ~ 5.013)	0.006	2.984 (1.340 ~ 6.645)	0.007
History of smoking	1.664 (0.853 ~ 3.244)	0.135		
AST/ALT	0.615 (0.307 ~ 1.233)	0.171		
TG, mmol/L	0.851 (0.714 ~ 1.014)	0.071		
TC, mmol/L	0.594 (0.432 ~ 0.816)	0.001	0.573 (0.404 ~ 0.814)	0.002
LDL, mmol/L	0.763 (0.520 ~ 1.119)	0.166		
HDL, mmol/L	0.256 (0.072 ~ 0.903)	0.034	0.641 (0.134 ~ 3.055)	0.576
White Blood Cell Count, *10^9^/L	1.219 (1.051 ~ 1.415)	0.009	1.218 (1.031 ~ 1.439)	0.020
C-reactive protein, mg/L	1.012 (0.994 ~ 1.031)	0.201		
NLR	1.091 (0.938 ~ 1.269)	0.260		
PLR	1.005 (0.999 ~ 1.010)	0.076		
ACEI/ARB	0.880 (0.330 ~ 2.348)	0.798		
β-blocker	2.526 (0.645 ~ 9.879)	0.183		
CCBs	2.144 (0.904 ~ 5.085)	0.084		
Statins	1.321 (0.659 ~ 2.651)	0.433		
LAD PCAT attenuation, HU	1.053 (1.002 ~ 1.106)	0.043	1.036 (0.983 ~ 1.092)	0.187
LCX PCAT attenuation, HU	1.046 (0.999 ~ 1.095)	0.055		
RCA PCAT attenuation, HU	1.069 (1.020 ~ 1.121)	0.006	1.069 (1.014 ~ 1.127)	0.013
LAD PCAT volume, mm3	1.000 (0.999 ~ 1.000)	0.286		
LCX PCAT volume, mm3	1.000 (0.999 ~ 1.000)	0.534		
RCA PCAT volume, mm3	1.000 (0.999 ~ 1.000)	0.209		
DS_max_	1.042 (1.022 ~ 1.062)	< 0.001	0.994 (0.961 ~ 1.028)	0.721
TPV	1.019 (1.012 ~ 1.027)	< 0.001	1.021 (1.008 ~ 1.035)	0.002

Notes: *OR* odds ratio, *95% CI* 95% confidence interval, *BMI* body mass index, *AST* aspartate aminotransferase, *ALT* alanine aminotransferase, *TG* triglycerides, *NLR* neutrophil-to-lymphocyte ratio, *PLR* platelet-to-lymphocyte ratio, *ACEI* angiotensin-converting enzyme inhibitors, *ARB* angiotensin receptor blockers, *β-blocker* beta-blocker, *PCAT* pericoronary adipose tissue, *LAD* left anterior descending artery, *LCX* left circumflex artery, *RCA* right coronary artery, *DS* diameter stenosis, *TPV* total plaque volume

### Discrimination

**Fig. 5:**
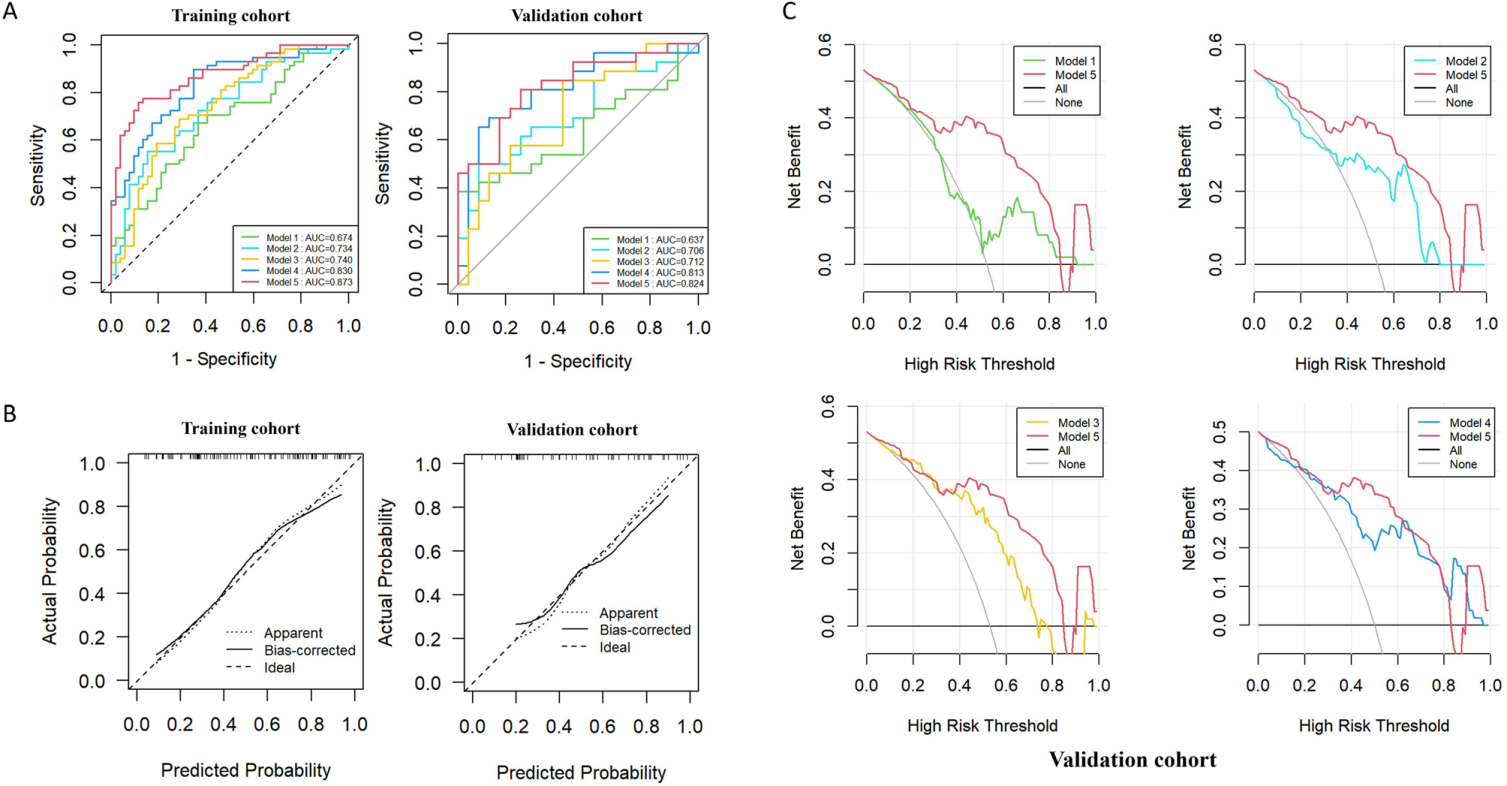
To assess the effectiveness of various models in identifying NAFLD patients with INOCA. **A** ROC Curves of All Models for the Training and Validation Cohorts; **B** Calibration Curves for the Training and Validation Cohorts of the Conbined Imaging-clinical Models; **C** DCA for the Validation Group Comparing Models 1–4 with Model 5. Model 1, PCATa model; Model 2, Radiomics model; Model 3, Clinical factors model; Model 4, Combined imaging model; Model 5, Combined imaging-clinical model

Figure 5 A presents the performance of the models in the training and validation cohorts, along with pairwise comparisons of the ROC curves. The discriminatory ability of the models was quantified by assessing the AUC, specificity, sensitivity, accuracy, positive predictive value (PPV), and negative predictive value (NPV), as summarized in Table 3. Model 2 exhibited superior diagnostic performance for INOCA in patients with NAFLD compared with Model 1, with AUCs of 0.734 (95% CI: 0.640–0.828) and 0.674 (95% CI: 0.574–0.779), respectively, in the training cohort and 0.706 (95% CI: 0.558–0.853) and 0.637 (95% CI: 0.478–0.797), respectively, in the validation cohort. Model 4 demonstrated greater discriminatory ability than Model 3, achieving an AUC of 0.830 (95% CI: 0.755–0.906) in the training cohort and 0.813 (95% CI: 0.687–0.939) in the validation cohort. Thus, the incorporation of clinical factors into Model 5 significantly improved its diagnostic performance. Model 5 achieved an AUC of 0.873 (95% CI: 0.808–0.937) in the training cohort and 0.824 (95% CI: 0.708–0.941) in the validation cohort. This model was superior to Models 1, 2, and 3 individually, demonstrating significantly improved incremental reclassification performance. Furthermore, the NRI and IDI metrics confirmed the superior predictive capability of Model 5 in both the training and validation cohorts (all *p* < 0.05) (Supplemental Table 2).

**Table 3:** Recognition ability of all models for patients with INOCA

	Training group						Validation group					
	AUC(95%CI)	SEN	SPE	ACC	PPV	NPV	AUC(95%CI)	SEN	SPE	ACC	PPV	NPV
Model 1	0.674(0.574–0.779)	0.707	0.500	0.609	0.612	0.605	0.637(0.478–0.797)	0.615	0.478	0.551	0.571	0.524
Model 2	0.734(0.640–0.828)	0.724	0.615	0.673	0.677	0.667	0.706(0.558–0.853)	0.654	0.522	0.592	0.607	0.571
Model 3	0.740(0.647–0.834)	0.741	0.635	0.691	0.694	0.688	0.712(0.565–0.860)	0.577	0.739	0.653	0.714	0.607
Model 4	0.830(0.755–0.906)	0.724	0.731	0.727	0.750	0.704	0.813(0.687–0.939)	0.731	0.696	0.714	0.731	0.696
Model 5	0.873(0.808–0.937)	0.776	0.731	0.755	0.763	0.745	0.824(0.708–0.941)	0.769	0.652	0.714	0.714	0.714

Notes: *INOCA* ischaemia with non-obstructive coronary arteries, *AUC* area under curve, *95%CI* 95% confidence interval, *SEN* sensitivity, *SPE* specificity, *ACC* accuracy, *PPV* positive predictive value, *NPV* negative predictive value

Model 1 = PCATa model; Model 2 = Radiomics model; Model 3 = Clinical factors model; Model 4 = Combined imaging model; Model 5 = Combined imaging-clinical model

### Calibration and clinical application

The calibration curves of the comprehensive model exhibited strong agreement between predicted and observed outcomes in both cohorts, indicating good calibration (Fig. 5 B). DCA further showed that Model 5 provided the greatest net clinical benefit across a wide range of threshold probabilities, suggesting its potential clinical applicability (Fig. 5 C).

## Discussion

In this study, we compared PCAT and clinical indicators between patients with NAFLD and INOCA and those without, and further evaluated the feasibility of identifying concurrent INOCA. RCA-PCATa and radiomic features were effective for distinguishing NAFLD with INOCA, with radiomics models outperforming the standalone PCATa model. Furthermore, the combination of imaging indicators (PCATa, radiomics, TPV) surpassed clinical factors, whereas integrating imaging and clinical factors achieved the highest discriminatory ability for INOCA diagnosis.

Although traditional diagnostic approaches focus on evaluating coronary artery stenosis, particularly through the assessment of obstructive disease, increasing evidence suggests that non-obstructive CAD also holds significant clinical relevance. Non-obstructive CAD, typically defined as coronary artery stenosis of less than 50%, can still result in ischemic symptoms and adverse cardiovascular outcomes due to underlying pathological mechanisms such as inflammation, plaque vulnerability, and microvascular dysfunction [5, 27]. Our study emphasizes the crucial role of inflammation in the development of INOCA, where systemic chronic inflammation, common in NAFLD, exacerbates coronary artery inflammation, thus contributing to ischaemia despite non-obstructive stenosis [8, 12]. PCAT, as a biomarker of vascular inflammation, reflects the dynamic pathological changes in atherosclerosis, with higher PCATa often indicating more severe inflammatory activity closely associated with the development and progression of CAD [28]. Our study found that patients with NAFLD exhibited significantly higher PCATa than patients without NAFLD, a finding that is consistent with that of previous studies, suggesting that elevated PCATa is a novel cardiovascular risk predictor in patients with NAFLD [12, 22], a trend that persists even in non-obstructive CAD. We further demonstrated that patients with NAFLD and INOCA have significantly higher LAD-PCATa and RCA-PCATa than those without, showing that patients with INOCA experience more pronounced coronary perivascular inflammation, likely because of lipid metabolism disorders, chronic inflammatory responses, and the critical role of PCAT in coronary artery pathology [8]. However, although increased LAD-PCATa was observed in patients with INOCA, our results revealed that only RCA-PCATa was an indicator for distinguishing patients with NAFLD and INOCA from those without, which was inconsistent with previous studies [22, 29]. This discrepancy may be attributed to the fact that prior research indicated that stenosis was more commonly observed in the LAD in patients with NAFLD, whereas no significant stenosis was found in the LAD in our cohort. In contrast, the RCA, because of its fewer branches and greater amount of pericoronary adipose tissue, can serve as a routine parameter reflecting overall coronary inflammation [24]. Moreover, RCA-PCAT may be more sensitive to systemic inflammatory changes in metabolic diseases such as NAFLD, possibly due to its relatively stable anatomical position and reduced susceptibility to motion artifacts. These factors may account for the greater predictive value of RCA-PCATa observed in our study. In addition, prior studies have suggested that RCA-PCATa values above − 76.3 HU are indicative of increased coronary inflammation and heightened cardiovascular risk [30]. While no universally accepted cutoff currently exists, our observation that NAFLD patients with INOCA exhibited significantly elevated RCA-PCATa supports its potential as a non-invasive biomarker for risk stratification. Rather than focusing on a single threshold, our results based on continuous RCA-PCATa values demonstrate a consistent association with coronary inflammation. These findings suggest that incorporating PCAT attenuation into routine CCTA interpretation could enhance risk stratification of NAFLD patients with non-obstructive stenosis, who might otherwise be underrecognized by traditional stenosis-based assessments.

In distinguishing patients with NAFLD and INOCA from those without INOCA, the radiomics model exhibited superior predictive performance compared with the PCAT model (training: AUC: 0.734 vs. 0.674; validation: AUC: 0.706 vs. 0.637). This may be because vascular inflammation induces irreversible changes in adipose tissue, such as PCAT fibrosis and microvascular remodeling [14, 15], which cannot be adequately captured by PCATa values alone. This advantage of the radiomics model may be because of its ability to extract high-dimensional features through quantitative analysis and pattern recognition of medical imaging data, which significantly enhances the characterization of tissue and lesion properties compared with PCATa, providing deeper diagnostic insights [31, 32]. Furthermore, it enhances capture tissue heterogeneity, inflammatory status, and plaque stability, offering a more comprehensive assessment of vascular pathology [33]. PCAT radiomic features have been used to explore the association between coronary artery inflammation and CAD occurrence in several studies [11, 34]. Zhang et al. [11] constructed a PCAT radiomics scoring model based on seven representative radiomic features and demonstrated that this model significantly outperformed conventional PCAT parameters in diagnosing CAD. Furthermore, a retrospective case–control study reported that PCAT radiomic features derived from CCTA provided higher predictive accuracy for acute coronary syndrome than traditional PCATa parameters [34]. These findings collectively highlight the complementary role of PCAT radiomics alongside conventional PCAT parameters, offering clinicians more precise diagnostic references and facilitating early intervention and timely treatment.

Thus, our study developed and evaluated a comprehensive imaging model that integrates PCAT radiomics and imaging indicators to assist in the diagnosis of INOCA in patients with NAFLD. The improved performance of the combined imaging model may be attributed to its ability to capture subtle tissue heterogeneity and spatial relationships within PCAT [11] while providing anatomical parameters, such as plaque characteristics, thereby enabling a more comprehensive evaluation of coronary artery plaques and inflammation. Furthermore, in our analysis, the combined imaging-clinical model showed the best discriminatory ability among all models. Clinical characteristics, such as sex, BMI, WBC count, and diabetes mellitus, provide the fundamental assessment of patient risk, whereas imaging parameters may improve diagnostic precision by capturing disease-specific features that are not readily detectable through clinical evaluation alone. This integrative approach could be particularly informative in patients with NAFLD, given that the distinct metabolic and pericoronary inflammatory environment associated with the disease might contribute to an underestimation of cardiovascular risk when relying solely on traditional risk scores. The findings of Li et al. [22] further support the use of this approach and demonstrated that the combination of clinical features, CTA parameters, and PCAT radiomics has potential to improve the risk prediction of stable CAD in patients with NAFLD. The development of a combined model may therefore offer a useful exploratory framework rather than a definitive diagnostic tool for assessing the likelihood of NAFLD progressing to CAD, particularly in individuals with non-obstructive CAD, and could help guide early monitoring and further investigation in future studies.

Despite these encouraging results, this study has several limitations that should be acknowledged. First, this was a single-center retrospective study with a relatively small sample size, which may have restricted the generalizability of the findings because no external validation data were available for confirmation. Future research should incorporate data from multiple centers with larger and more diverse populations to enhance the applicability and enable a more comprehensive analysis. Second, all patients in this study underwent imaging using the same CT scanning protocol. The mechanisms underlying the influence of variations in CT equipment settings on the radiomic parameters remain unclear. Further studies are necessary to evaluate the robustness of radiomic features across different imaging protocols and scanners. Third, the model was not compared with traditional risk prediction tools (e.g., Framingham or ASCVD), which may have limited the demonstration of the incremental value of radiomics; future studies will incorporate more comprehensive clinical data to allow validation and direct comparison. Fourth, although this study focused on radiomic feature extraction from RCA-PCAT, future research should expand to include PCAT features from other coronary artery segments. This approach would provide a more comprehensive assessment of inflammation-related changes and improve our understanding of the full spectrum of vascular inflammation.

## Conclusion

PCAT radiomics demonstrated potential value in identifying INOCA in patients with NAFLD. Integrating PCAT radiomic features with imaging indicators may provide an effective approach to improve the diagnosis of INOCA in this population, outperforming clinical factors alone in our study. The combined clinical-imaging model achieved the highest discriminatory performance in distinguishing patients with INOCA from those without, supporting its potential clinical utility in patients with NAFLD.

## Supplementary Information

Supplementary Material 1.

Supplementary Material 2.

## Data Availability

The datasets used and analyzed during the current study are available from the corresponding author on reasonable request.

## References

[1] Powell EE, Wong VW, Rinella M. Non-alcoholic fatty liver disease. Lancet. 2021;397(10290):2212–24. 33894145 10.1016/S0140-6736(20)32511-3

[2] Duell PB, Welty FK, Miller M, et al. Nonalcoholic fatty liver disease and cardiovascular risk: a scientific statement from the American Heart Association. Arterioscler Thromb Vasc Biol. 2022;42(6):e168–85. 35418240 10.1161/ATV.0000000000000153

[3] Simon TG, Roelstraete B, Alkhouri N, et al. Cardiovascular disease risk in paediatric and young adult non-alcoholic fatty liver disease. Gut. 2023;72(3):573–80. 36522149 10.1136/gutjnl-2022-328105

[4] Gholoobi A, Gifani M, Gholoobi A, et al. Relationship between the prevalence and severity of non-alcoholic fatty liver disease and coronary artery disease: findings from a cross-sectional study of a referral center in Northeast Iran. JGH Open. 2022;6(5):330–7. 35601123 10.1002/jgh3.12746 PMC9120894

[5] Hjort M, Eggers KM, Lakic TG, et al. Biomarker concentrations and their temporal changes in patients with myocardial infarction and nonobstructive compared with obstructive coronary arteries: results from the PLATO trial. J Am Heart Assoc. 2023;12(1):e27466. 10.1161/JAHA.122.027466 PMC9973579 36565198

[6] Bairey MC, Pepine CJ, Walsh MN, et al. Ischemia and no obstructive coronary artery disease (INOCA): developing Evidence-Based therapies and research agenda for the next Decade. Circulation. 2017;135(11):1075–92. 28289007 10.1161/CIRCULATIONAHA.116.024534 PMC5385930

[7] Chan K, Wahome E, Tsiachristas A, et al. Inflammatory risk and cardiovascular events in patients without obstructive coronary artery disease: the ORFAN multicentre, longitudinal cohort study. Lancet. 2024;403(10444):2606–18. 38823406 10.1016/S0140-6736(24)00596-8 PMC11664027

[8] Prati F, Marco V, Paoletti G, et al. Coronary inflammation: why searching, how to identify and treat it. Eur Heart J Suppl. 2020;22(Suppl E):E121–4. 32523455 10.1093/eurheartj/suaa076 PMC7270901

[9] Francque SM, van der Graaff D, Kwanten WJ. Non-alcoholic fatty liver disease and cardiovascular risk: pathophysiological mechanisms and implications. J Hepatol. 2016;65(2):425–43. 27091791 10.1016/j.jhep.2016.04.005

[10] Antonopoulos AS, Sanna F, Sabharwal N, et al. Detecting human coronary inflammation by imaging perivascular fat. Sci Transl Med. 2017;9(398):eaal2658. 28701474 10.1126/scitranslmed.aal2658

[11] Zhang C. Coronary heart disease evaluation using PCAT radiomics model based on coronary CT angiography and pericoronary adipose tissue. Medicine. 2024;103(42):e39936. 39432597 10.1097/MD.0000000000039936 PMC11495773

[12] Ichikawa K, Miyoshi T, Osawa K, et al. Association between higher pericoronary adipose tissue attenuation measured by coronary computed tomography angiography and nonalcoholic fatty liver disease: a matched case-control study. Medicine. 2021;100(34):e27043. 34449489 10.1097/MD.0000000000027043 PMC8389939

[13] Knuuti J, Wijns W, Saraste A, et al. 2019 ESC guidelines for the diagnosis and management of chronic coronary syndromes. Eur Heart J. 2020;41(3):407–77. 31504439 10.1093/eurheartj/ehz425

[14] Crewe C, An YA, Scherer PE. The ominous triad of adipose tissue dysfunction: inflammation, fibrosis, and impaired angiogenesis. J Clin Invest. 2017;127(1):74–82. 28045400 10.1172/JCI88883 PMC5199684

[15] Oikonomou EK, Antoniades C. The role of adipose tissue in cardiovascular health and disease. Nat Rev Cardiol. 2019;16(2):83–99. 30287946 10.1038/s41569-018-0097-6

[16] Dong X, Li N, Zhu C, et al. Diagnosis of coronary artery disease in patients with type 2 diabetes mellitus based on computed tomography and pericoronary adipose tissue radiomics: a retrospective cross-sectional study[J]. Cardiovasc Diabetol. 2023;22(1):14. 36691047 10.1186/s12933-023-01748-0 PMC9869509

[17] Oikonomou EK, Williams MC, Kotanidis CP, et al. A novel machine learning-derived radiotranscriptomic signature of perivascular fat improves cardiac risk prediction using coronary CT angiography. Eur Heart J. 2019;40(43):3529–43. 31504423 10.1093/eurheartj/ehz592 PMC6855141

[18] Ren Z, Wen D, Xue R, et al. Nonalcoholic fatty liver disease is associated with myocardial ischemia by CT myocardial perfusion imaging, independent of clinical and coronary CT angiography characteristics. Eur Radiol. 2023;33(6):3857–66. 36571601 10.1007/s00330-022-09306-0

[19] Shimokawa H, Suda A, Takahashi J, et al. Clinical characteristics and prognosis of patients with microvascular angina: an international and prospective cohort study by the coronary vasomotor disorders international study (COVADIS) group. Eur Heart J. 2021;42(44):4592–600. 34038937 10.1093/eurheartj/ehab282 PMC8633728

[20] Beltrame JF, Tavella R, Jones D, et al. Management of ischaemia with non-obstructive coronary arteries (INOCA)[J]. BMJ. 2021;375:e60602. 10.1136/bmj-2021-060602 34836873

[21] Yang H, Teng H, Luo P, et al. The role of left ventricular hypertrophy measured by echocardiography in screening patients with ischaemia with non-obstructive coronary arteries: a cross-sectional study. Int J Cardiovasc Imaging. 2023;39(9):1657–66. 37237153 10.1007/s10554-023-02879-x

[22] Li N, Dong X, Zhu C, et al. Association study of NAFLD with pericoronary adipose tissue and pericardial adipose tissue: diagnosis of stable CAD patients with NAFLD based on radiomic features. Nutr Metab Cardiovasc Dis. 2025;35(1):103678. 39107221 10.1016/j.numecd.2024.06.020

[23] Nishihara T, Miyoshi T, Ichikawa K, et al. Association of oxidized low-density lipoprotein in nonalcoholic fatty liver disease with high-risk plaque on coronary computed tomography angiography: a matched case-control study. J Clin Med. 2022;11(10):2838. 35628964 10.3390/jcm11102838 PMC9144234

[24] Oikonomou EK, Marwan M, Desai MY, et al. Non-invasive detection of coronary inflammation using computed tomography and prediction of residual cardiovascular risk (the CRISP CT study): a post-hoc analysis of prospective outcome data. Lancet. 2018;392(10151):929–39. 30170852 10.1016/S0140-6736(18)31114-0 PMC6137540

[25] Ma R, van Assen M, Ties D, et al. Focal pericoronary adipose tissue attenuation is related to plaque presence, plaque type, and stenosis severity in coronary CTA. Eur Radiol. 2021;31(10):7251–61. 33860371 10.1007/s00330-021-07882-1 PMC8452552

[26] Oikonomou EK, Desai MY, Marwan M, et al. Perivascular fat attenuation index stratifies cardiac risk associated with high-risk plaques in the CRISP-CT study. J Am Coll Cardiol. 2020;76(6):755–7. 32762910 10.1016/j.jacc.2020.05.078

[27] Mehta PK, Huang J, Levit RD, et al. Ischemia and no obstructive coronary arteries (INOCA): a narrative review. Atherosclerosis. 2022;363:8–21. 36423427 10.1016/j.atherosclerosis.2022.11.009 PMC9840845

[28] Mancio J, Oikonomou EK, Antoniades C. Perivascular adipose tissue and coronary atherosclerosis. Heart. 2018;104(20):1654–62. 29853488 10.1136/heartjnl-2017-312324

[29] Ichikawa K, Miyoshi T, Nakashima M, et al. Prognostic value of pericoronary adipose tissue attenuation in patients with non-alcoholic fatty liver disease with suspected coronary artery disease. Heart Vessels. 2022;37(12):1977–84. 35672527 10.1007/s00380-022-02107-x

[30] van Diemen PA, Bom MJ, Driessen RS, et al. Prognostic value of RCA pericoronary adipose tissue CT-attenuation beyond high-risk plaques, plaque volume, and ischemia. JACC Cardiovasc Imaging. 2021;14(8):1598–610. 33958312 10.1016/j.jcmg.2021.02.026

[31] Shang J, Guo Y, Ma Y, et al. Cardiac computed tomography radiomics: a narrative review of current status and future directions. Quant Imaging Med Surg. 2022;12(6):3436–53. 35655815 10.21037/qims-21-1022 PMC9131324

[32] Kolossvary M, Kellermayer M, Merkely B, et al. Cardiac computed tomography radiomics: a comprehensive review on radiomic techniques. J Thorac Imaging. 2018;33(1):26–34. 28346329 10.1097/RTI.0000000000000268

[33] Li Y, Huo H, Liu H, et al. Coronary CTA-based radiomic signature of pericoronary adipose tissue predict rapid plaque progression. Insights Imaging. 2024;15(1):151. 38900243 10.1186/s13244-024-01731-7 PMC11189889

[34] Jing M, Xi H, Sun J, et al. Differentiation of acute coronary syndrome with radiomics of pericoronary adipose tissue. Br J Radiol. 2024;97(1156):850–8. 38366613 10.1093/bjr/tqae032 PMC11027295

