# Supplementary material for: Radiomics analysis of pericoronary adipose tissue for detecting ischaemia with non-obstructive coronary arteries in NAFLD patients
Source: BMC Cardiovasc Disord. 2025 Nov 12;25:803. doi: 10.1186/s12872-025-05292-5 (PMC12613792; doi:10.1186/s12872-025-05292-5)
Supplement: Supplementary file 1 — Supplementary Material 1. [file 12872_2025_5292_MOESM1_ESM.docx]

**Rad-score formula:**

Rad-score=2.7754−1.4971×(Cluster Prominence)−1.3420×(Low Gray Level Run Emphasis)

The predicted probability of INOCA was calculated as:


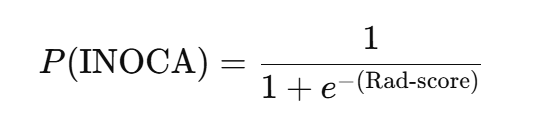


| **Supplemental Table3. Logistic regression coefficients for the radiomics signature** | | |
| --- | --- | --- |
| Variable | Coefficient (β) | Feature type |
| Intercept (β0) | 2.7754 | — |
| Cluster Prominence (β1) | –1.4971 | GLCM |
| Low Gray Level Run Emphasis (β2) | –1.3420 | GLRLM |
| **Notes: All radiomic features were standardized using z-score normalization. A higher Rad-score indicates a higher estimated probability of INOCA, while the negative coefficients imply that increases in Cluster Prominence and Low Gray Level Run Emphasis reduce the Rad-score and thus lower the predicted probability.** | | |
