# Supplementary material for: Radiomics analysis of pericoronary adipose tissue for detecting ischaemia with non-obstructive coronary arteries in NAFLD patients
Source: BMC Cardiovasc Disord. 2025 Nov 12;25:803. doi: 10.1186/s12872-025-05292-5 (PMC12613792; doi:10.1186/s12872-025-05292-5)
Supplement: Supplementary file 2 — Supplementary Material 2. [file 12872_2025_5292_MOESM2_ESM.docx]

| **Supplemental Table1** Baseline Characteristics According to the Training cohort and testing cohort | | | | |
| --- | --- | --- | --- | --- |
|  | All patients  N=159 | Training group  N=110 | Validation group  N=49 | P value |
| **Clinical characteristics** |  |  |  |  |
| Age(y) | 63.04 ± 11.44 | 62.82 ± 12.46 | 63.55 ± 8.84 | 0.673 |
| Gender (male),n(%) | 86 (54.09) | 65 (59.09) | 21 (42.86) | 0.058 |
| BMI, kg/m2 | 26.67 (24.40, 29.11) | 26.57 (24.48, 28.82) | 26.76 (24.30, 29.79) | 0.778 |
| Hypertension, n (%) | 92 (57.86) | 64 (58.18) | 28 (57.14) | 0.902 |
| Diabetes,n (%) | 58 (36.48) | 42 (38.18) | 16 (32.65) | 0.540 |
| History of smoking, n (%) | 54 (33.96) | 41 (37.27) | 13 (26.53) | 0.187 |
| **Lipids** |  |  |  |  |
| AST, U/L | 23.00 (19.00, 33.00) | 22.50 (18.25, 33.00) | 23.00 (20.00, 32.00) | 0.568 |
| ALT, U/L | 24.00 (16.50, 37.00) | 25.00 (17.00, 37.00) | 20.00 (16.00, 33.00) | 0.295 |
| AST/ALT | 1.05 (0.77, 1.31) | 0.98 (0.75, 1.27) | 1.14 (0.85, 1.36) | 0.071 |
| TC, mmol/L | 4.81 (4.09, 5.38) | 4.79 (4.10, 5.38) | 4.82 (4.09, 5.38) | 0.840 |
| TG, mmol/L | 2.05 (1.42, 3.06) | 2.13 (1.42, 3.14) | 1.79 (1.43, 2.44) | 0.451 |
| LDL-C, mmol/L | 2.70 (2.11, 3.31) | 2.70 (2.11, 3.34) | 2.70 (2.11, 3.22) | 0.851 |
| HDL-C, mmol/L | 1.04 (0.89, 1.21) | 1.04 (0.89, 1.22) | 1.06 (0.94, 1.20) | 0.613 |
| **Inflammatory markers** |  |  |  |  |
| White Blood Cell Count,*109/L | 6.63 (5.68, 8.25) | 6.67 (5.76, 8.06) | 6.56 (5.54, 8.48) | 0.981 |
| C-reactive protein, mg/L | 3.67 (1.27, 7.44) | 3.54 (1.28, 7.44) | 3.67 (1.21, 7.44) | 0.675 |
| NLR | 2.47 (1.78, 3.37) | 2.41 (1.87, 3.43) | 2.49 (1.68, 3.25) | 0.430 |
| PLR | 111.63 (87.82, 144.99) | 110.18 (88.24, 149.64) | 114.83 (86.77, 139.01) | 0.839 |
| **PCAT attenuation, HU** |  |  |  |  |
| LAD | -79.90 ± 6.65 | -80.44 ± 6.05 | -78.69 ± 7.77 | 0.125 |
| LCX | -73.21 ± 7.09 | -73.16 ± 6.98 | -73.33 ± 7.41 | 0.885 |
| RCA | -78.45 ± 7.58 | -78.40 ± 7.65 | -78.58 ± 7.51 | 0.893 |
| **PCAT volume, mm3** |  |  |  |  |
| LAD | 1974.26 ± 446.19 | 1972.73 ± 439.78 | 1977.70 ± 464.87 | 0.949 |
| LCX | 1313.83 ± 450.23 | 1344.90 ± 457.74 | 1244.08 ± 429.28 | 0.193 |
| RCA | 2236.83 ± 570.09 | 2214.62 ± 579.30 | 2286.70 ± 551.40 | 0.463 |
| **DS, n (%)** |  |  |  |  |
| LAD | 0.00 (0.00, 28.00) | 0.00 (0.00, 24.50) | 12.00 (0.00, 30.00) | 0.095 |
| LCX | 0.00 (0.00, 15.00) | 0.00 (0.00, 15.00) | 0.00 (0.00, 0.00) | 0.565 |
| RCA | 16.00 (0.00, 28.00) | 15.50 (0.00, 27.00) | 19.00 (0.00, 29.00) | 0.744 |
| DS_max_ | 22.00 (4.00, 39.50) | 20.50 (0.00, 43.50) | 24.00 (12.00, 35.00) | 0.425 |
| **TPV, mm^3^** | 55.48 (6.50, 96.23) | 53.09 (0.00, 101.57) | 61.93 (27.36, 86.66) | 0.652 |
| Notes: BMI, Body Mass Index; AST, Aspartate Aminotransferase; ALT , Alanine Aminotransferase; TC, total cholesterol; TG, triglycerides; LDL, low-density lipoprotein; HDL, high-density lipoprotein; NLR, Neutrophil-to-Lymphocyte Ratio; PLR, Platelet-to-Lymphocyte Ratio; ACEI, angiotensin-converting enzyme inhibitors; ARB, angiotensin receptor blockers; β-blocker, Beta-Blocker; PCAT, Pericoronary Adipose Tissue; DS, diameter stenosis; TPV, Total Plaque Volume; DS, Diameter Stenosis; LAD, Left Anterior Descending Artery; LCX, Left Circumflex Artery; RCA, Right Coronary Artery. | | | | |

| **Supplemental Table2** Comparison of Discriminant and Reclassification Capacities of Each Model for Diagnosing NAFLD with INOCA | | | | | | | | | | |
| --- | --- | --- | --- | --- | --- | --- | --- | --- | --- | --- |
|  | Training group | | | | | Validation group | | | | |
| Comparison | P value | NRI | P value | IDI | P value | P value | NRI | P value | IDI | P value |
| Model 1 vs Model 4 | 0.002 | 0.410 | <0.001 | 0.214 | <0.001 | 0.012 | 0.473 | 0.003 | 0.220 | <0.001 |
| Model 2 vs Model 4 | 0.018 | 0.350 | <0.001 | 0.175 | <0.001 | 0.165 | 0.338 | 0.034 | 0.143 | 0.007 |
| Model 1 vs Model 5 | 0.002 | 0.427 | <0.001 | 0.302 | <0.001 | 0.016 | 0.565 | <0.001 | 0.356 | <0.001 |
| Model 2 vs Model 5 | 0.018 | 0.425 | <0.001 | 0.263 | <0.001 | 0.161 | 0.517 | 0.003 | 0.278 | <0.001 |
| Model 3 vs Model 5 | <0.001 | 0.373 | <0.001 | 0.227 | <0.001 | 0.102 | 0.217 | <0.001 | 0.181 | 0.002 |
| Model 4 vs Model 5 | 0.002 | 0.133 | 0.002 | 0.087 | 0.001 | 0.796 | 0.141 | 0.003 | 0.136 | 0.011 |
| Notes: In comparisons labeled “Model X vs Model Y,” Model X (on the left) represents the older model, while Model Y (on the right) represents the newer model. Model 1= PCATa model Model 2= Radiomics model;; Model 3= Clinical factors model; Model 4= Combined imaging model; Model 5= Combined imaging-clinical model. Abbreviations: INOCA, Ischaemia with Non-obstructive Coronary Arteries; AUC, area under curve; NRI, Net Reclassification Improvement; IDI, Integrated Discrimination Improvement. | | | | | | | | | | |
